# Birefringence of Thin Uniaxial Polymer Films Estimated Using the Light Polarization Ellipse

**DOI:** 10.3390/polym14051063

**Published:** 2022-03-07

**Authors:** Mihai Postolache, Dan Gheorghe Dimitriu, Cristina Delia Nechifor, Simona Condurache Bota, Valentina Closca, Dana Ortansa Dorohoi

**Affiliations:** 1 Faculty of Automatic Control and Computer Engineering, Gheorghe Asachi Technical University, 700050 Iasi, Romania; mpostol@ac.tuiasi.ro; 2 Faculty of Physics, Alexandru Ioan Cuza University, 700506 Iasi, Romania; vclosca@gmail.com (V.C.); ddorohoi@uaic.ro (D.O.D.); 3 Faculty of Machine Manufacturing and Industrial Management, “Gheorghe Asachi” Technical University, 700050 Iasi, Romania; cd13_nechifor@yahoo.com; 4 Faculty of Sciences and Environment, Dunarea de Jos University, 800201 Galati, Romania; scondurache@ugal.ro; 5 Department of Science, Eudoxiu Hurmuzachi National College, 725400 Radauti, Romania

**Keywords:** linear birefringence of polymer stretched foils, polarized light, polarization ellipse, poly(vinyl alcohol)

## Abstract

A simple method for determining the linear birefringence of the thin layers based on the determination of the orientation of the polarization ellipse of totally polarized light is proposed and it is applied to PVA thin foils. Theoretical notions and the experimental procedure are described. The linear birefringence of polymer thin foils with different degrees of stretching is determined and the applicability of the method is discussed.

## 1. Introduction

The uniaxial anisotropic media are characterized by two main refractive indices, *n_o_* and *n_e_*, named ordinary and extraordinary refractive indices and measured with linearly polarized radiations having their electric field perpendicular, respectively parallel to the optical axis of the anisotropic medium. The difference:

(1) $$\Delta n\left(\lambda \right)=n_{e}\left(\lambda \right)-n_{o}\left(\lambda \right)$$

is named linear birefringence. It is a material dependent and dispersive parameter. For the transparent media, the linear birefringence decreases with an increase in light wavelength [1,2]. The birefringence of the inorganic crystals is usually smaller than that of stretched polymer foils or of liquid crystalline layers. The last two materials have the advantage that their birefringence can be modified by external field of forces [1,2].

There are some methods for determining both linear [3,4,5,6,7,8] and circular [9,10,11,12,13] birefringence of the anisotropic layers, based on the compensation of the optical path length [3,4,5,6,7,8,9,10,11,12,13,14,15] or interferometric means [3,8,10]. For thick anisotropic layers the method based on channeled spectra [3,10,12] is usually applied. Other methods were developed for different types of anisotropic layers, as for example liquid crystals [16,17,18].

A very simple method for determining the linear birefringence of thin anisotropic layers is described in this paper and it is applied to the stretched polymer foils. Poly(vinyl alcohol) (PVA) is used to obtain the foils for birefringence determination in our experiments.

PVA is a white granular polymer soluble in hot water and insoluble in the most organic solvents and in cold water [19].

PVA films cast from water solutions have high tensile strength, tear resistance, and mechanical stability. They are resistant to oils, grease, or solvents and are impermeable for most gases. PVA films have multiple applications due to their environmental friendliness, easy processing, and low costs [19,20]. The properties of the PVA polymer films can be easily modified by external actions [21,22,23].

PVA films are widely used in various applications in industry or medicine due to their low costs of fabrication and simple manufacturing. They are transparent in visible range, have low weight, and are characterized by elasticity and flexibility [24,25,26,27].

Polarizers and optical compensators are made of polymers and used in liquid crystals displays (LCDs) or in organic light-emitting diodes (OLEDs) displays [25,26,28]. The knowledge on the optical properties (transparency, birefringence, and dichroism) of polymer films is very important for obtaining high performance displays (wide viewing angle, high contrast ratio, low color shift etc.) [27,29,30,31].

In medicine, PVA foils are used as biomaterials due to their degradability, in drug delivery, in tissue replacement for improving or correcting human organs [32,33,34,35].

Due to some of its properties (high flexibility of polymer chains, mechanical strength, durability, very good film formability, covalent crosslinking capacity, self-healing capability, chemical stability, long-term temperature and pH stability, non-toxicity, biodegradability, biocompatibility, easy processability, excellent transparency), PVA is widely used in the composition of electroactive polymer materials. Thus, physically crosslinked PVA-H_2_SO_4_ or PVA-H_3_PO_4_ hydrogels are used as electrolytes for the flexible supercapacitors [36,37,38]. For different applications, the above-mentioned hydrogels are often combined with conductive polymers as polyaniline (PANI) [39,40,41,42,43,44], poly(vinyl pyrrolidone) (PVP) [45], poly(acrylic acid) (PAA) [46,47,48,49], polypropylene (PP) [50], poly(3,4-ethylenedioxythiophene)-poly(styrenesulfonate) (PEDOT:PSS) [51], or cellulose [52,53]. By adding TiO_2_ nanoparticles to the combination PVA + PANI, an increase of the actuation strain of the nanofiber webs was observed [54], while the combination PVA + PANI with graphitic carbon nitride (g-C_3_N_4_) proved to be very sensitive for chloride ion detection [55]. Functional network hydrogels were fabricated from PVA + PVP and a dynamic ferric cross-linked cellulose nanocrystals (CNCs-Fe^3+^) network, acting as flexible and wearable strain sensors for human healthcare monitoring or sensory skin in soft robotics [45]. Hydrogels based on PVA + PAA can be applied for biomimetics actuators, artificial muscles, and electroactive reconfigurable lens [49], but also for drug release [46,47], due to high sensitivity to both pH and temperature. PVA + PP can be used in actuators in response to temperature and humidity, and also in soft robotics, as soft artificial muscle and self-adapted smart manipulator [50]. Electroactive biomimetic artificial muscle based on PVA + PEDOT: PSS was developed [51]. By adding cellulose to PVA, nontoxic, soft, and electroactive hydrogel was obtained, with applications in artificial muscles developing [52] and drug release [53]. In combination with carbon nanotubes or carboxylated multiwalled nanotubes, PVA hydrogels are used as ultrafiltration membranes [56,57], as well as actuators with potential applications ranging from microswitches to artificial muscles, robotics, optical displays, prosthetic devices, microscopic pumps, and anti-vibration systems [58].

The birefringence of polymers derives from the asymmetry of their molecular structures. The intrinsic optical anisotropy of PVA caused by partial order of their chains can be increased by stretching. The stretching-induced anisotropy is added to the intrinsic one. The colored and stretched PVA foils become dichroic and can change the light spectral composition [59,60,61,62,63].

A very convenient and simple method to characterize the anisotropy of polymer films by their birefringence is described in [3]. There, the dispersion of birefringence in the visible range is determined based on channeled spectra obtained for the studied polymer films, fixed between crossed polarizers. This method is very precise and allows measurements for all components of light in a single experiment. The birefringence can be determined by using compensators (e.g., Babinet compensator used in our laboratory [4,5,6,7,8,14,15]), but the experiment asks for monochromatic radiations for which the compensatory device must be standardized.

The interferometric measurements are also used for the birefringence estimation. One measures each of the principal refractive indices (*n_o_* and *n_e_* for uniaxial layers) from the relative shift of the central fringes in the two interference fields, one mobile and one fixed. The mobile system of fringes is determined by the optical path difference between two transparent layers—the polymer film introduced in the measuring beam and an isotropic layer with the refractive index value between the values of *n_o_* and *n_e_*. Two identical polarizing filters are introduced on the paths of the two beams of interferometer, giving the mobile system of fringes. In this method, the interferometer must be standardized for each monochromatic radiation and the central fringe can be evidenced only by using white light [6,7].

## 2. Theoretical Notions

The total polarized light changes its polarization state function on the birefringence and the thickness of the anisotropic material in which it propagates.

The usual mode for the representation of polarized light propagation inside an anisotropic medium is to consider the linearly polarization light as being equivalent to two components linearly polarized on two perpendicular directions and taking into consideration the phase difference, Δ*ψ*, introduced between them in the propagation process [1,2]. The phase difference is determined by the birefringence, Δ*n*, and by the distance of propagation through the anisotropic medium, *l*, as relation (2) shows.

(2) $$\Delta \psi =\frac{2\pi }{\lambda }\Delta n\left(\lambda \right)l$$

The phase difference depends on the spectral composition of light by the wavelength of radiation, *λ*, and also by the dispersion of the material birefringence.

Let us consider the principal system of coordinates Oabc (in which the refractive index has only two values) of a uniaxial anisotropic layer with optical axis oriented along the Oc axis and the light propagation along the Ob principal axis. This choice simplifies the theory exposed below.

**Figure 1:**
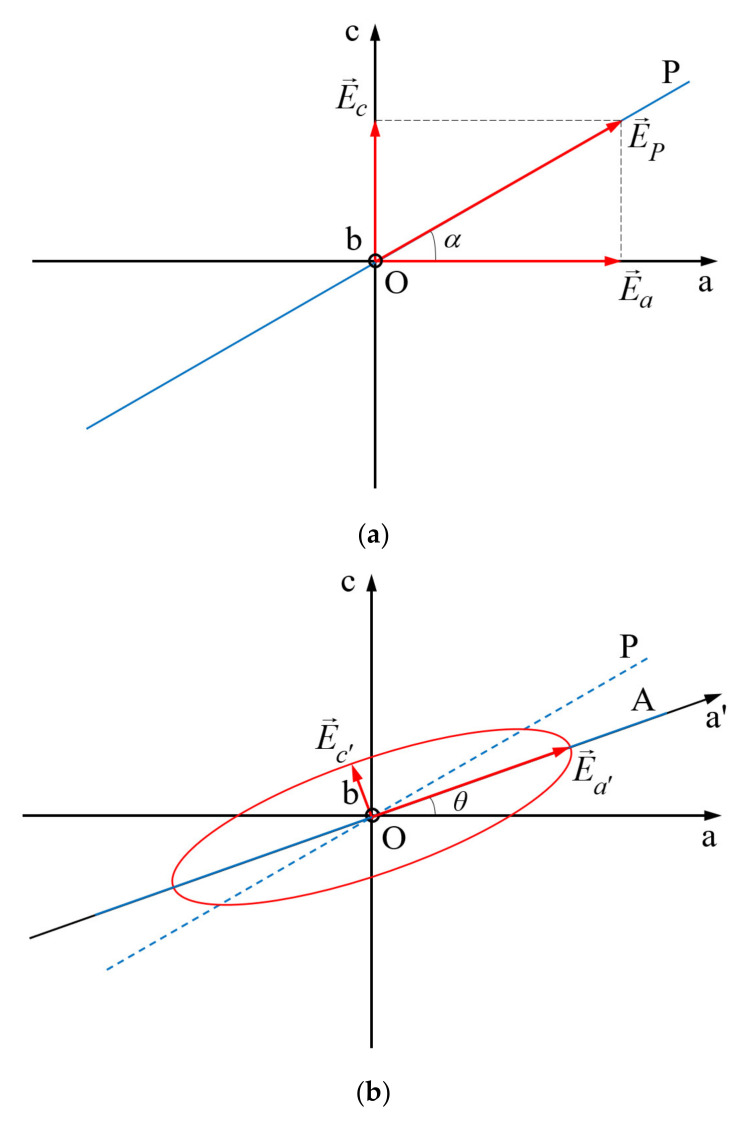
Relative positions of the transmission directions of the polarizing filters P and A (drawn with blue lines) in the principal plane Oac of anisotropic material ((**a**) before the anisotropic layer, (**b**) after the anisotropic layer).

Let be the azimuth angle-*α*-the angle between the axis Oa of the anisotropic medium and the transmission direction of a polarizing filter P (see Figure 1).

The polarizing filter P transforms the incident randomly polarized light into a linear polarized light with its electric field amplitude parallel to the transmission direction of the filter. The real parts of the electric field components acting on axes Oa and Oc are:

(3) $$\begin{array}{l} e_{a}=E_{a}\cos\omega t\\ e_{c}=E_{c}\cos\left(\omega t+\Delta \psi \right) \end{array}$$

The phase difference between the two components was translated to component acting on the principal axis Oc for simplicity of the calculations. In relation (3), the magnitudes of the two components are projections of the electric field amplitude after the polarizer P, *E_P_*, on the principal axes Oa and Oc. One can write:

(4) $$\begin{array}{c} E_{a}=E_{P}\cos\alpha \\ E_{c}=E_{P}\sin\;\alpha \end{array}$$

By eliminating the time between the components (3) of the light electric field, one obtains the equation of the polarization ellipse (5) described by the amplitude of the light electric field in its rotation around the propagation direction. The electric field amplitude has its origin on the light propagation direction.

(5) $${\left(\frac{e_{a}}{E_{P}\cos\alpha }\right)}^{2}+{\left(\frac{e_{c}}{E_{P}\sin\alpha }\right)}^{2}-2\frac{e_{a}e_{c}}{E_{P}^{2}\cos\alpha \sin\alpha }\cos\Delta \psi =\sin^{2}\Delta \psi$$

Equation (5) represents an ellipse in Oac plane, which degenerates into a line for the cases Δ*ψ* = *m*π, with *m* = 1, 2, 3, …, or in a circle for *E_a_* = *E_c_* and $\Delta \psi =\frac{2m+1}{2}\pi$, with *m* = 0, 1, 2, … When Δ*ψ* = 2*mπ*, with *m* = 1, 2, 3, …, the emergent light from the anisotropic layer is linearly polarized and keeps its azimuth, *α*, and for Δ*ψ* = (2*m* + 1)π, with *m* = 0, 1, 2, …, the emergent light is also linear polarized but has the azimuth π – *α*.

When $\Delta \psi =\frac{2m+1}{2}\pi$, with *m* = 0, 1, 2, …, and *E_a_* ≠ *E_c_*, the ellipse described by Equation (5) has its semiaxes parallel to the principal axes of the anisotropic layer, being left or right polarized in function on the *m* values. When $m\pi <\Delta \psi <\frac{2m+1}{2}\pi$, with *m* = 0, 1, 2, the polarization ellipse has its semi axes rotated with an angle *θ* relative to the principal axes of the anisotropic layer.

The angles *α* (corresponding to the orientation of the electric field in the incident polarized light onto the anisotropic layer) and *θ* (giving the orientation of the polarization ellipse relative to the principal axis Oa of the anisotropic layer) can be used in determining the birefringence of the anisotropic layer.

Let be a rotation of the polarizing filter A with angle *θ* around the light propagation direction. The dependence of the light components in the two orientations (*O*ac and *O*a’c’ in Figure 1) of the principal axes of coordinates is described by Equation (6):

(6) $$\begin{array}{c} e_{a}=e_{a’}\cos\theta -e_{c’}\sin\theta \\ e_{c}=e_{c’}\cos\;\theta +e_{a’}\sin\;\theta \; \end{array}$$

By introducing (6) in (5), and taking into consideration relations (4), one obtains:

(7) $$\begin{array}{l} e_{a’}^{2}\left(\frac{\cos^{2}\theta }{E_{P}^{2}\cos^{2}\alpha }+\frac{\sin^{2}\theta }{E_{P}^{2}\sin^{2}\alpha }+2\frac{\sin\theta \cos\theta }{E_{P}^{2}\sin\alpha \cos\alpha }\cos\Delta \psi \right)+\\ +e_{c’}^{2}\left(\frac{\sin^{2}\theta }{E_{P}^{2}\cos^{2}\alpha }+\frac{\cos^{2}\theta }{E_{P}^{2}\sin^{2}\alpha }-2\frac{\sin\theta \cos\theta }{E_{P}^{2}\sin\alpha \cos\alpha }\cos\Delta \psi \right)-\\ -2e_{a’}e_{c’}e_{a’}^{2}\left(\frac{\sin\theta \cos\theta }{E_{P}^{2}\cos^{2}\alpha }-\frac{\sin\theta \cos\theta }{E_{P}^{2}\sin^{2}\alpha }+\frac{\cos^{2}\theta -\sin^{2}\theta }{E_{P}^{2}\sin\alpha \cos\alpha }\cos\Delta \psi \right)=\sin^{2}\Delta \psi \end{array}$$

The polarization ellipse is reported to the axes *O*a’ and *O*c’ if the third term in (7) is null, in the condition (8):

(8) $$\cos\Delta \psi =\frac{\tan2\theta }{\tan2\alpha }$$

The equation of the ellipse (7) reported to axes *O*a’ and *O*c’ rotated with angle *θ* around the propagation direction, in the condition (8), becomes:

(9) $${\left(\frac{e_{a’}}{E_{a’}}\right)}^{2}+{\left(\frac{e_{c’}}{E_{c’}}\right)}^{2}=1$$

The flux densities of the radiations are measured after an analyzer (A) (see Figure 1), having its transmission direction parallel to the new semiaxes of the ellipse (9). The values of the flux densities measured when the transmission direction of A is parallel to *O*a’ and to *O*c’ are proportional with the square of its semi-axes. When one obtains the maximum and minimum of the flux density by rotating the transmission direction of the analyzer around the light propagation direction, one can determine the axes *O*a’ and *O*c’ of the polarization ellipse (9). The angle *θ* is now known.

Based on relations (8) and (2), one can write:

(10) $$\Delta n=\frac{\lambda }{2\pi l}\arccos\frac{\tan2\theta }{\tan2\alpha }$$

The relative error of birefringence estimation by using the above equation was calculated by using the logarithm method, in the next measurement accuracy conditions: Δ*λ* = 0.6 nm, Δ*l* = 1 μm, Δ*θ* = Δ*α* = 0.01°. The obtained result, 2.15%, can be improved by increasing the accuracy of anisotropic layer thickness measurement, which is the main source of error (approximate 93% from the total relative error).

The birefringence can be determined for thin anisotropic layer, as it is described in the experimental part if the layer thickness is known.

## 3. Experimental

As anisotropic layer, PVA thin films were used. They were prepared using PVA 20% solution in distilled water, stirred for 5 h at 80 °C. This solution was casted onto a glass substrate and left to dry for 3 days at 27 °C, until a solid film was obtained. A rubbing action was applied before the complete drying of the PVA film, extensively described in [21]. The polymer film was stretched under gentle heating (an incandescent lamp of 70 W was placed at 15 cm in front of the stretching device).

The degree of stretching, *γ*, is determined by the ratio of the semiaxes of an ellipse in which a circle, initially drawn on the surface of the unstretched polymer foil, degenerates after the stretching process.

**Figure 2:**
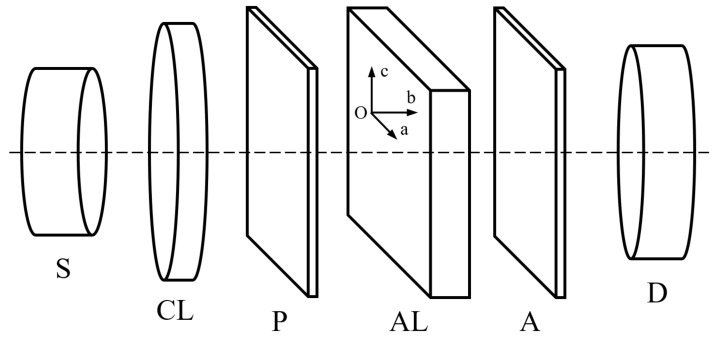
Schematic device used to determine the main axes of the polarizing ellipse. (S—light source, CL—converging lens in collimator arrangement, P and A identical polarizing filters, AL anisotropic layer, D detector).

In order to obtain conditions exposed in theoretical notions, one uses a device consisting of two polarizing filters having between them the anisotropic layer (AL), as shown in Figure 2. The polarizing filter P transforms the randomly polarized light into a linearly polarized light with its electric field parallel to the transmission direction of the polarizer.

The monochromatic light can be obtained from white light with an interferential filter. However, our experimental determinations were made with yellow light (*λ* = 589.3 nm) emitted by a sodium lamp. The diameter of the collimated light beam illuminating the sample is 6 mm. Initially the two polarizing filters are crossed, and light does not pass through them. When the anisotropic layer (a polymer stretched layer of PVA with the dimensions 15 mm × 15 mm, inserted into a circular mount with the diameter of 10 mm) is introduced between the two polarizers, light changes its polarization state and passes through the device. The luminous flux is measured by the light detector D, which is a Si-based photodetector with amplifier, manufactured by PHYWE and having the spectral range 390–1150 nm.

The polymer stretched foil is a uniaxial anisotropic layer having its optical axis parallel to the stretching direction. The light propagation is perpendicular to the layer surface and parallel with one principal axis of anisotropic medium, let it be Ob axis.

The polarizer filters have known transmission directions marked on their settings. Initially one choses an azimuth angle, *α*, by rotating the polarizing filter P around the propagation direction (Ob). For a given *α*, one rotates the second polarizing filter around the same direction, to obtain the maximum flux density (*φ_a’_*) and to identify the high semi axis of the polarization ellipse. One notes the value of the angle *θ*. The minimum of the flux density (*φ_c’_*) corresponding to the small axis of the polarization ellipse is obtained after a supplemental rotation around the propagation direction with 90°.

The rotation angles were measured with digital angle gauge inclinometers, rigidly fixed on the mounts of the polarizing filters P and A, respectively. The accuracy of angle measurement is 0.01°, which determines a contribution to the total relative error of birefringence estimation of around 2% (1% for *θ* and 1% for *α*).

The emergent flux from the analyzer A is measured using a circuit with a diode having high sensitivity in the spectral range to which the monochromatic light belongs. In this way the maximum and minimum of the flux density in the emergent beam are easily evidenced.

## 4. Results and Discussion

The proposed method can be applied in the labs where other devices for estimating birefringence are absent (interferometers, compensators, or polarizing microscopes) in order to estimate with enough precision (a relative error of around 2% in the conditions of using common (inexpensive) measuring instruments) the birefringence of the thin anisotropic layers, in particular of polymer-stretched foils.

In the cases when the phase difference between the ordinary and extraordinary rays is higher than 90 degrees (for high degrees of stretching or for high thickness of the layer), a non-determination bonded to the periodicity of cosine function can appear. Even the thickness of the layer can be precisely estimated, however, the birefringence cannot be exactly determined.

In this situation, a supplemental experiment can be carried out. Two small pieces having close values of thickness, cut from the same stretched film are used in this experiment. They are assembled with the optical axes perpendicular in a single anisotropic foil and used as AL in the device described above. In this arrangement the ordinary ray from one anisotropic thin piece becomes extraordinary ray in the second one. In this case the total phase difference between the ordinary and extraordinary rays emerging from the anisotropic layer, expressed in (11), is proportional to the difference in the thicknesses $L_{1}$ and $L_{2}$ of the polymer pieces composing AL, measured on the light propagation direction.

(11) $$\Delta \psi =\frac{2\pi }{\lambda }\Delta n\left(L_{1}-L_{2}\right)$$

The results of the measurements are given in the Table 1, Table 2 and Table 3. Each experimental value was measured ten times and the averaged values are presented in the tables.

In Table 1, the results of measurements for a PVA foil rubbed as it was described in [14] are noted, showing the intrinsic birefringence of the polymer foils. In this table, the angles *α* and *θ* and the flux densities measured along the semi axes of the rotated ellipse are noted, for a given PVA polymer foil. The values of the phase differences for various azimuth angles were computed with formula (8) and the birefringence can be computed using relation (10) for a single polymer foil, or relation (11) when the AL is composed by two polymer foils with crossed optical axes. The average value of the phase difference from the last column of Table 1 gives a value of birefringence of about 0.0023 for a thickness of the layer of 50 µM. The value of the linear birefringence computed based on data from Table 1 corresponds with the value determined using a Babinet compensator, by the method described in [15] (Δ*n* = 0.018 for *L* = 1300 μm and taking into consideration the variation of the birefringence with the change of the PVA film thickness [15]).

The rubbing action add supplemental small birefringence to the intrinsic one of the unstretched foil. The same conclusion resulted also from the experiments organized in [14].

**Figure 3:**
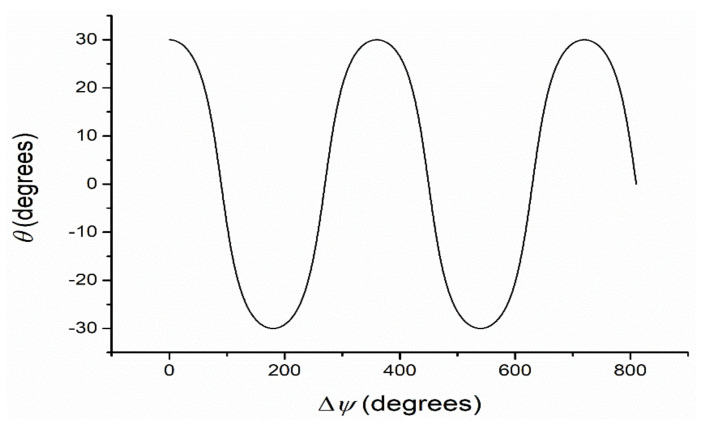
Computed dependence of angle *θ* vs. phase difference Δ*ψ* between the ordinary and extraordinary rays in AL (*α* = 30 degrees).

The phase difference, Δ*ψ*, depends, as it results from (2), on both the linear birefringence and the thickness of the anisotropic layer. When it becomes higher than 90 degrees, the angle *θ* is also higher than 90 degrees and the polarizing ellipse has its highest axis in the second dial of Oac plane. The computed dependence of the angle *θ* on the phase difference introduced by AL between the ordinary and extraordinary rays is illustrated in Figure 3.

From Figure 3 the results show that for more periodic values of the phase difference one obtains the same values for the angle *θ* giving the inclination of the high semi axes of the polarization ellipse, due to the periodicity of cosine function.

Having in view the periodicity of cosine function, the high values of the phase difference do not offer information about the multiple of 2π for which the experiment gives the same values of the angle *θ*. So, one must use a procedure to diminish the optical path length by using two plates having perpendicular optical axes and with small difference between their thicknesses.

For a degree of stretching higher than 1.50, the birefringence was estimated using two pieces cut from the same stretched foil assembled with their stretching directions perpendicular. In this case, the values of the birefringence must be computed with relation (11).

The experimental data obtained for one PVA foil with the degree of stretching of about *γ* = 1.90 are given in Table 2. A value of 0.0166804 has been experimentally obtained (see Table 2). The experimental data contained in Table 2 and Table 3 show that the polymer foils birefringence increases in stretching process, as was already confirmed in previous experiments [3,15,21]. To verify the result from Table 3 another two foils were cut from the initial PVA foil, and the experiment for determining the semi axes of the polarization ellipse was repeated. The same data were obtained.

The birefringence measured with a Babinet compensator [3] showed similar values as those measured by the method described here.

## 5. Conclusions

The linear birefringence of thin foils (like polymer ones) can be determined using a simple device consisting of two polarizing filters with an anisotropic thin layer between them, and a photodiode for the luminous flux measuring. The device is illuminated in parallel beam with monochromatic radiation emitted by a lamp or obtained with monochromatic filter.

For small values of the linear birefringence and small foil thickness, one uses a single sample cut from a stretched foil. Having in view the periodicity of cosine function, for high values of the phase differences one can use two crossed polymer films with their optical axes perpendicular, to reduce the phase difference between the ordinary and extraordinary rays crossing the anisotropic layer.

The proposed method is very simple, cost and time effective, and requires usual laboratory devices. The samples do not require a special treatment and they can have any dimensions, however large enough to be included in a mount. For sample’s thickness values larger than 0.1 mm (this value depends on the birefringence of the sample), two samples with close thicknesses are required. By using multiple light sources (with different value of the emitted light wavelength) or white light in combination with multiple filters, the dispersion of the birefringence can be also recorded. However, the method of channeled spectra [3] is more efficient (in terms of time consuming) for this.

The described method can be applied also for determining the birefringence of transparent electroactive polymers. The knowledge of birefringence could open the way for new applications of electroactive polymers in electrooptic devices.

## Figures and Tables

**Table 1:** Phase difference between the ordinary and extraordinary radiations, Δ*ψ*, determined by PVA polymer foil (*L* = 50 µM).

*α* (Degrees)	*θ* (Degrees)	*φ_a’_* (a.u.)	*φ_c’_* (a.u.)	tan2*α*	tan2*θ*	Δ*ψ* (Degrees)	Δ*n* ^1^
10	3.4	0.974	0.027	0.364	0.119	70.88	0.002305
20	7.8	0.988	0.113	0.839	0.279	70.29	0.002301
25	10.6	0.846	0.154	1.192	0.388	71.00	0.002324
30	15.2	0.789	0.211	1.732	0.587	70.20	0.002298
35	20.7	0.732	0.268	2.747	0.882	71.28	0.002334
40	31.7	0.685	0.315	5.671	1.997	69.38	0.002714
43	38.9	0.665	0.334	14.300	4.625	71.12	0.002328

^1^ The average value of the birefringence is 0.002315 ± 0.000057, calculated after eliminating the value corresponding to *α* = 40° (intrinsic birefringence of PVA rubbed foil), the relative error being 2.46%.

**Table 2:** Phase difference between the ordinary and extraordinary radiations, Δ*ψ*, determined by PVA polymer foil (*L*_1_ = 1.255 mm, *L*_2_ = 1.260 mm, Δ*L* = 5 μM, *γ* = 1.90).

*α* (Degrees)	*θ* (Degrees)	*φ_a’_* (a.u.)	*φ_c’_* (a.u.)	tan2*α*	tan2*θ*	Δ*ψ* (Degrees)	Δ*n* ^1^
10	6.45	0.982	0.018	0.364	0.229	51.00	0.0166969
20	13.9	0.933	0.067	0.839	0.527	51.10	0.0167296
25	18.4	0.901	0.098	1.192	0.748	51.12	0.0167361
30	23.8	0.858	0.142	1.732	1.095	50.78	0.0162481
35	30.0	0.841	0.159	2.747	1.732	50.92	0.0166704
40	37.1	0.821	0.179	5.671	3.534	51.45	0.0168442
43	41.8	0.813	0.187	14.300	8.999	51.43	0.0168376

^1^ The average value of the birefringence is 0.0166804 ± 0.0001264, the relative error being 0.76%.

**Table 3:** Phase difference between the ordinary and extraordinary radiations, Δ*ψ*, determined by PVA polymer foil (*L*_1_ = 1.376 mm, *L*_2_ = 1.371 mm, Δ*L* = 5 μM, *γ* = 2.60).

*α* (Degrees)	*θ* (Degrees)	*φ_a’_* (a.u.)	*φ_c’_* (a.u.)	tan2*α*	tan2*θ*	Δ*ψ* (Degrees)	Δ*n* ^1^
10	1.8	0.97	0.02	0.364	0.070	78.91	0.0258343
20	4.2	0.89	0.12	0.839	0.148	79.84	0.0261387
25	5.9	0.83	0.17	1.192	0.209	79.90	0.0261584
30	8.4	0.76	0.24	1.732	0.302	79.96	0.0261780
35	13.0	0.68	0.31	2.747	0.488	79.78	0.0261909
40	22.3	0.62	0.38	5.671	0.986	79.99	0.0261878
43	34.2	0.59	0.40	14.300	2.526	79.83	0.0261355

^1^ The average value of the birefringence is 0.0261177 ± 0.0000809, the relative error being 0.31%.

## Data Availability

The data presented in this study are available on request from the corresponding author.
